# Wearable Finger Pulse Oximetry for Continuous Oxygen Saturation Measurements During Daily Home Routines of Patients With Chronic Obstructive Pulmonary Disease (COPD) Over One Week: Observational Study

**DOI:** 10.2196/12866

**Published:** 2019-06-06

**Authors:** Joren Buekers, Jan Theunis, Patrick De Boever, Anouk W Vaes, Maud Koopman, Eefje VM Janssen, Emiel FM Wouters, Martijn A Spruit, Jean-Marie Aerts

**Affiliations:** 1 Health Unit Flemish Institute for Technological Research (VITO) Mol Belgium; 2 Measure, Model & Manage Bioresponses Department of Biosystems KU Leuven Leuven Belgium; 3 Centre for Environmental Sciences Hasselt University Diepenbeek Belgium; 4 Department of Research and Education Centre of Expertise for Chronic Organ Failure (CIRO) Horn Netherlands; 5 Department of Respiratory Medicine Maastricht University Medical Centre Maastricht Netherlands; 6 Rehabilitation Research Center (REVAL), Biomedical Research Institute (BIOMED) Faculty of Rehabilitation Sciences Hasselt University Diepenbeek Belgium; 7 School of Nutrition and Translational Research in Metabolism (NUTRIM) Maastricht University Medical Centre Maastricht Netherlands

**Keywords:** COPD, oxygen saturation, finger pulse oximeter, wearable sensor, nocturnal desaturation, telemonitoring

## Abstract

**Background:**

Chronic obstructive pulmonary disease (COPD) patients can suffer from low blood oxygen concentrations. Peripheral blood oxygen saturation (SpO_2_), as assessed by pulse oximetry, is commonly measured during the day using a spot check, or continuously during one or two nights to estimate nocturnal desaturation. Sampling at this frequency may overlook natural fluctuations in SpO_2_.

**Objective:**

This study used wearable finger pulse oximeters to continuously measure SpO_2_ during daily home routines of COPD patients and assess natural SpO_2_ fluctuations.

**Methods:**

A total of 20 COPD patients wore a WristOx_2_ pulse oximeter for 1 week to collect continuous SpO_2_ measurements. A SenseWear Armband simultaneously collected actigraphy measurements to provide contextual information. SpO_2_ time series were preprocessed and data quality was assessed afterward. Mean SpO_2_, SpO_2_ SD, and cumulative time spent with SpO_2_ below 90% (CT90) were calculated for every (1) day, (2) day in rest, and (3) night to assess SpO_2_ fluctuations.

**Results:**

A high percentage of valid SpO_2_ data (daytime: 93.27%; nocturnal: 99.31%) could be obtained during a 7-day monitoring period, except during moderate-to-vigorous physical activity (MVPA) (67.86%). Mean nocturnal SpO_2_ (89.9%, SD 3.4) was lower than mean daytime SpO_2_ in rest (92.1%, SD 2.9; *P*<.001). On average, SpO_2_ in rest ranged over 10.8% (SD 4.4) within one day. Highly varying CT90 values between different nights led to 50% (10/20) of the included patients changing categories between desaturator and nondesaturator over the course of 1 week.

**Conclusions:**

Continuous SpO_2_ measurements with wearable finger pulse oximeters identified significant SpO_2_ fluctuations between and within multiple days and nights of patients with COPD. Continuous SpO_2_ measurements during daily home routines of patients with COPD generally had high amounts of valid data, except for motion artifacts during MVPA. The identified fluctuations can have implications for telemonitoring applications that are based on daily SpO_2_ spot checks. CT90 values can vary greatly from night to night in patients with a nocturnal mean SpO_2_ around 90%, indicating that these patients cannot be consistently categorized as desaturators or nondesaturators. We recommend using wearable sensors for continuous SpO_2_ measurements over longer time periods to determine the clinical relevance of the identified SpO_2_ fluctuations.

## Introduction

Chronic obstructive pulmonary disease (COPD) is a highly prevalent lung disease that is characterized by persistent airflow limitation due to a mixture of obstructive bronchiolitis and emphysema [1]. Morbidity and mortality of COPD are high and still increasing [2], leading COPD to become the third-leading cause of death worldwide by 2030 [3]. COPD patients can suffer from low blood oxygen concentrations due to gas exchange abnormalities [1]. Hypoxemia during the night (ie, nocturnal desaturation) is also common in patients with COPD [4-6] due to nocturnal alveolar hypoventilation and ventilation-perfusion mismatching [7]. Hypoxemia can worsen with increasing disease severity [8]. Furthermore, it has been shown that hypoxemia is associated with lower exercise tolerance; decreased quality of life; increased risk for exacerbations, defined as “a sustained worsening of the patient's condition, from the stable state and beyond normal day-to-day variations” [9]; and higher risks of death [8].

The presence of hypoxemia can be assessed by invasive blood gas analyses. A noninvasive method to assess peripheral blood oxygen saturation (SpO_2_) is pulse oximetry. Spot check SpO_2_ measurements with thresholds of 88%-92% have been suggested for the detection of hypoxemia [1,10,11]. In telemonitoring applications, daily SpO_2_ spot checks are used to raise alerts for exacerbations when SpO_2_ spot check values drop below predefined SpO_2_ thresholds [12]. Nocturnal desaturation is usually defined as having an SpO_2_ value below 90% for more than 30% of the time in bed, measured during one [6,13-16] or two nights [5,17-20].

The current SpO_2_ monitoring strategies do not take into account natural fluctuations in SpO_2_. Sampling SpO_2_ with such a low time frequency or during such a short time period may thus lead to classification errors (ie, hypoxemic or nonhypoxemic and nocturnal desaturator or nondesaturator) or false alerts in telemonitoring applications [12]. Wearable finger pulse oximeters provide the possibility to collect SpO_2_ data at higher time frequencies and over longer time periods. This makes it possible to assess and account for oxygen saturation fluctuations in patients with COPD. Therefore, the objective of this study was to use wearable finger pulse oximeters to continuously measure SpO_2_ during daily home routines of COPD patients and assess natural SpO_2_ fluctuations. We hypothesized that significant natural SpO_2_ fluctuations are present within and between multiple days and nights, which may lead to classification errors (ie, nocturnal desaturator or nondesaturator) or false alerts in telemonitoring applications.

## Methods

### Study Design and Participants

**Figure 1:**
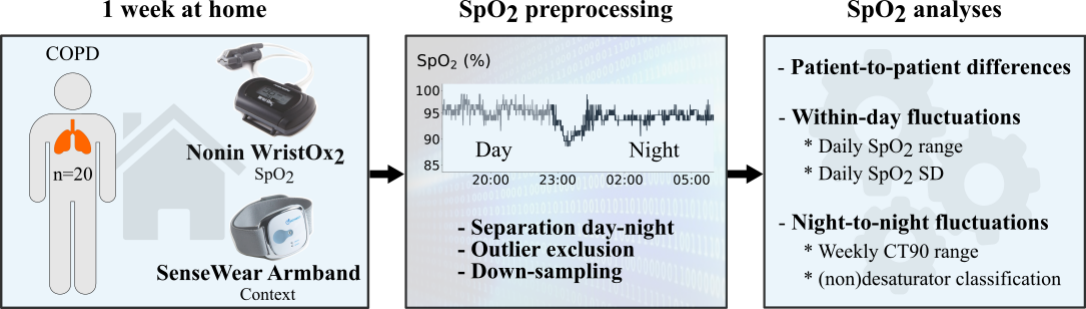
General overview of the applied methods. COPD: chronic obstructive pulmonary disease; SpO_2_: peripheral blood oxygen saturation; CT90: cumulative time spent with SpO_2_ below 90%.

Figure 1 provides a general overview of the methods applied in this single-center, 1-week observational study. COPD patients at Global Initiative for Chronic Obstructive Lung Disease stages II-IV (GOLD II-IV) were recruited at the Centre of Expertise for Chronic Organ Failure (CIRO), a COPD treatment center located in Horn, the Netherlands, during a standard baseline assessment prior to pulmonary rehabilitation [21]. The target sample size was set a priori to 20 patients. COPD patients were eligible to enroll in the study based on the following criteria: (1) clinically stable (ie, no exacerbation in the past 4 weeks), (2) no rollator use, and (3) no long-term oxygen therapy. Patients that were interested in participating were called a few days after the baseline assessment to schedule a home visit for the delivery of the wearable sensors. During this visit, the functioning of the sensors and data acquisition protocol were explained, written informed consent was obtained, and a new visit was planned for collection of the sensors at the end of the study period. During the 7-day study period, which took place before the start of pulmonary rehabilitation, two phone calls were made to resolve potential technical difficulties. Demographics, oxygen partial pressure in arterial blood (PaO_2_), postbronchodilator pulmonary function data (ie, forced expiratory volume in 1 second [FEV1], forced vital capacity [FVC], and transfer factor for carbon monoxide [TLCO]), 6-minute walking distance (6MWD), and COPD assessment test (CAT) results were collected during the standard baseline assessment at CIRO. The study was approved by the Medical Research Ethics Committees United (MEC-U) (study approval number: NL58079.100.16) in the Netherlands and executed between December 2016 and April 2018.

### Wearable Sensors and Protocol

Continuous SpO_2_ measurements were performed for 1 week using a wearable finger pulse oximeter: WristOx_2_ 3150 (Nonin Medical). Nonin oximeters have frequently been used for home monitoring of patients with COPD [12] and the WristOx_2_ 3150 model complies with the International Organization for Standardization (ISO) standards ISO 10993-1 and ISO 80601-2-61. The manufacturer reports an accuracy of ±2% for SpO_2_ measurements [22]. Sampling frequency was fixed at 1 Hz. Only the cumulative measurement time was visible for the participants. SpO_2_ values were not shown on the WristOx_2_ display to prevent patients from changing their behavior when deviating SpO_2_ values would occur. Every participant received three WristOx_2_ devices to deal with the limited battery life (ie, 48 hours of continuous measurements with one WristOx_2_). Participants were instructed to wear the WristOx_2_ on the index finger of their nondominant hand every night and as much as possible during the day, depending on their daily routines and comfort of wearing the finger clip. Raw data were stored on the internal memory of the WristOx_2_ and downloaded at the end of the week using nVISION software, version 6.4.0.10 (Nonin Medical).

Simultaneously, actigraphy was performed with the SenseWear Armband (BodyMedia) for obtaining contextual information about physical activity levels and when the participants were lying down and/or asleep. The SenseWear Armband is a multisensory triaxial accelerometer, combining accelerometry with measurements of heat flux, galvanic skin response, and skin temperature. Based on these measurements, the armband provides information about, for example, energy expenditure (EE), expressed as metabolic equivalent of task (MET), or steps taken, while also indicating when the wearer is lying down and sleeping, at a standard sampling time of 1 sample per minute. Measurements of MET were used for classifying physical activity levels, whereas the indications of lying down and sleeping were used to separate daytime measurements from nocturnal measurements, as further described below. The SenseWear Armband has been shown to be accurate for measurements of both physical activity [23,24], except when using a rollator [24], and sleep estimations [25]. Patients were asked to wear the armband on the left upper arm, except when there was contact with water (eg, when taking a shower). Battery life of one SenseWear Armband was sufficient for continuous 24-hour measurements with a 1-minute sampling time during the whole week. Data were stored on the internal memory and downloaded at the end of the week using the BodyMedia SenseWear 8.1 software (BodyMedia).

### Data Preprocessing

**Figure 2:**
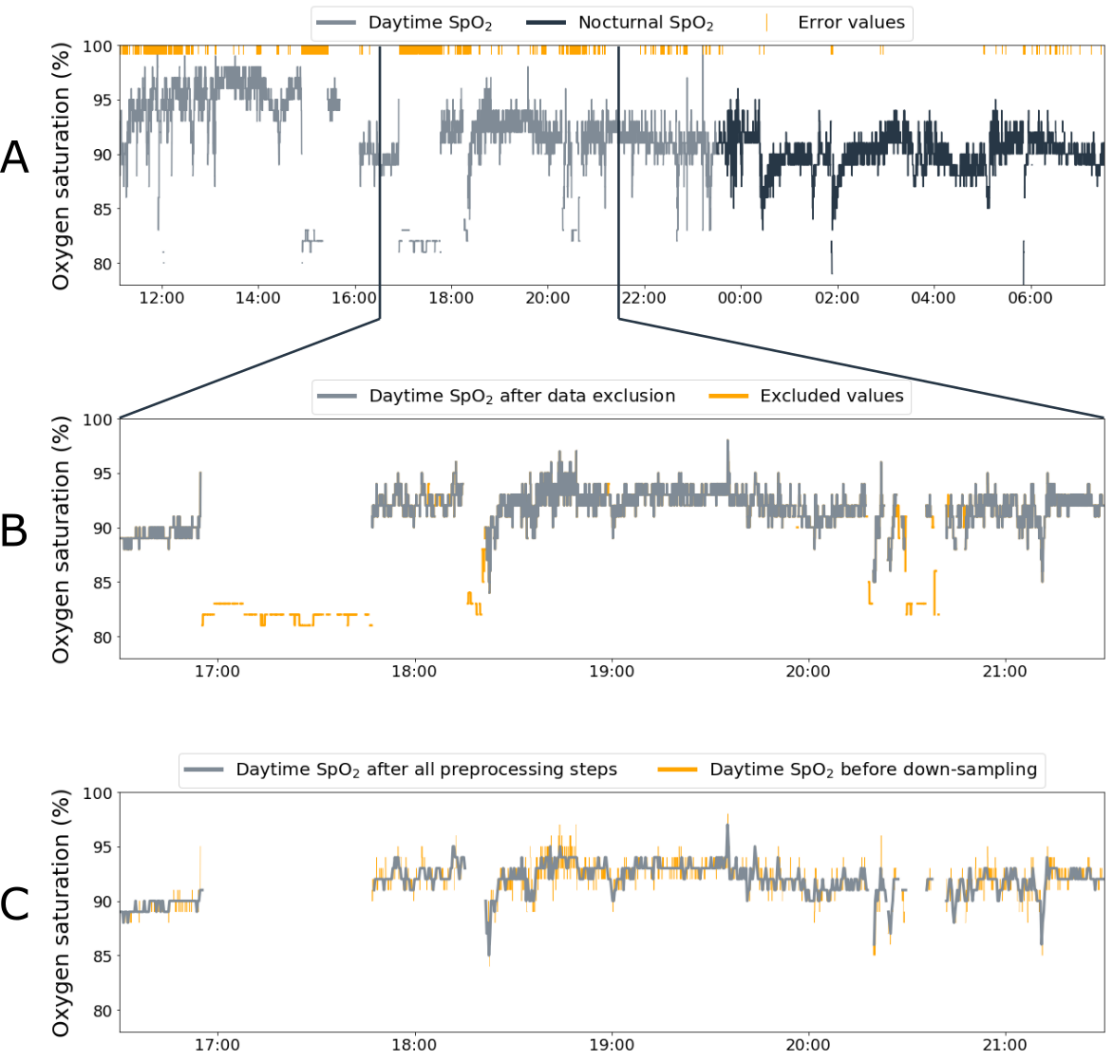
Visualization of the different preprocessing steps. Panel A shows all original data, containing error values, that are divided into daytime and nocturnal data. Panel B zooms in on the effect of data exclusion on a specific part of daytime peripheral blood oxygen saturation (SpO_2_) data. Panel C zooms in on the effect of down-sampling and interpolating on the same part of daytime SpO_2_ data.

Figure 2 visualizes the preprocessing steps. The SenseWear Armband indications about lying down and sleep were used to determine the time of going to bed in the evening and the time of getting out of bed in the morning. These time stamps were used to divide SpO_2_ data into nocturnal and daytime data (see Figure 2A). Only full-night nocturnal SpO_2_ measurements were retained for further analyses and daytime SpO_2_ measurements were retained if there was at least one hour of SpO_2_ measurements during that day. One hour of daytime measurements was considered sufficient to examine whether significant fluctuations occurred in SpO_2_ values during the day. Previous studies examining intraday fluctuations only examined measurements of one hour or less [26-29]. No days or nights had to be excluded due to lacking SenseWear Armband measurements. Days with at least one hour of measurements in both the afternoon and evening were used for the comparison between afternoon (ie, 13:00-18:00) and evening (ie, 18:00-going to bed) SpO_2_ values. No comparison was performed between SpO_2_ values in the morning (ie, before 13:00) and the afternoon and evening, because SpO_2_ measurements were often not performed before 13:00 (see Multimedia Appendix 1).

The raw SpO_2_ data contained error values (ie, the number 500 was provided in the raw data file) when measurements were considered invalid by the algorithms of the WristOx_2_ manufacturer (ie, orange dashes in Figure 2A). However, close inspection of the time series indicated that invalid data (eg, sudden low values) surrounded these error values. A data-cleaning algorithm was developed to exclude these invalid SpO_2_ values as follows. First, small blocks of data (ie, less than 20 samples) between error values were excluded. Second, bigger blocks of data (ie, between 20 and 100 samples) between error values were excluded, only when the mean SpO_2_ value of this data block was deviating more than 6% from the mean SpO_2_ value of the full day or night under consideration: both steps are shown in Figure 2B. Thorough visual and raw data examination of both valid and invalid data led to the choice of this 6% threshold for excluding invalid blocks of data (eg, Figure 2B: around 17:00-18:00). Third, remaining outliers were excluded by down-sampling the data (see Figure 2C). Autocorrelation analyses indicated that a sampling time of 20 seconds was appropriate (see Multimedia Appendix 2) and all data were thus down-sampled by taking the median SpO_2_ value of each consecutive 20-second block. By taking the median value, the effect of outliers was excluded. As a last step, small gaps of SpO_2_ data (ie, 3 or fewer missing samples) were filled using linear interpolation (see Figure 2C). After application of the data-cleaning algorithm, all data were visually checked to ensure valid SpO_2_ values were retained, while invalid values were removed.

### Data Analyses

The SenseWear Armband indications about lying down and sleep were used to calculate the total night sleeping time (TNST) (ie, sum of all minutes indicated as sleep), wake time after sleep onset (WASO) (ie, sum of all minutes spent awake during the time in bed, after the first onset of sleep), and sleep efficiency (Seff) (ie, the ratio between TNST and time in bed) for every separate night. Weekly averages of TNST, WASO, and Seff were calculated to describe the sleep quality of the included patients.

Daytime SpO_2_ data were divided into daytime data during rest (EE≤1.5 MET, while the patient was still awake), during low-intensity physical activities (LIPAs) (1.5 MET<EE≤3 MET), and during moderate-to-vigorous intensity physical activities (MVPAs) (EE>3 MET). Data quality of the continuous SpO_2_ measurements was assessed for both nocturnal and daytime data based on the amount of valid data (ie, excluding error values and cleaned values). Furthermore, the effect of physical activity on the amount of valid data was examined by comparing data quality during MVPA with data quality during rest and LIPA (EE≤3 MET).

Mean SpO_2_, SpO_2_ SD, and cumulative time spent with SpO_2_ below 90% (CT90) were calculated for every separate (1) day, (2) day in rest, and (3) night. Hereafter, the weekly average and weekly range (ie, the difference between the maximum and minimum value over the different days or nights of the same patient) of these features were calculated for every patient.

Intraday and intranight fluctuations were quantified as the weekly average of the standard deviation of the SpO_2_ measurements. The range of SpO_2_ values during the day in rest, calculated as the difference between the maximum and minimum SpO_2_ values of that day in rest, was determined to indicate how much spot-check values in telemonitoring applications could differ depending on the moment of the measurement. Only daytime SpO_2_ in rest was considered because only at these moments could spot checks have been performed in a telemonitoring application. In addition, the difference between mean SpO_2_ values in rest in the afternoon (ie, between 13:00 and 18:00) and in the evening (ie, between 18:00 and going to bed) was calculated for every day separately to examine differences in SpO_2_ baseline levels during the day.

Night-to-night and day-to-day SpO_2_ fluctuations were quantified as the weekly ranges of the three features (ie, mean SpO_2_, SpO_2_ SD, and CT90). Furthermore, we examined how many of the included patients changed category between nocturnal desaturator and nondesaturator.

### Statistical Analysis

Patient characteristics, weekly averages, and weekly ranges were summarized for all patients as mean and SD. Paired-sample *t* tests were used to test for differences between weekly averages of nocturnal and daytime SpO_2_ in rest. Pearson correlations assessed the relationship between mean SpO_2_ and patient characteristics, intraday SpO_2_ fluctuations, or intranight SpO_2_ fluctuations. A *P* value of <.05 was considered statistically significant. All analyses were carried out in Jupyter Notebooks (Project Jupyter) [30] using the Python 3.5 programming language (Python Software Foundation) [31].

## Results

### Participants

A total of 21 out of 41 patients that were approached accepted study participation. One patient suffered from an exacerbation before the start of the 7-day study period and was excluded. This finally led to the inclusion of 20 patients (14 males, 70%; 6 females, 30%) with moderate (8/20, 40%), severe (10/20, 50%), or very severe (2/20, 10%) COPD. General demographics, postbronchodilator lung function, resting arterial blood gases, 6MWD, CAT score, and sleep-quality characteristics are summarized in Table 1.

**Table 1.** Characteristics of the 20 included patients with moderate-to-very severe COPD^a^.

Characteristics	Mean (SD)
Age (years)	63 (8)
Body mass index (kg/m²)	26 (4)
Forced expiratory volume in 1 second (L)	1.4 (0.5)
Forced expiratory volume in 1 second (% predicted)	48 (15)
Forced vital capacity (L)	3.8 (1.1)
Forced vital capacity (% predicted)	97 (20)
Transfer factor for carbon monoxide (% predicted)	50 (16)
Partial pressure of oxygen (kPa)	8.7 (1.7)
Partial pressure of carbon dioxide (kPa)	5.2 (0.6)
6-minute walking distance (m)	411 (59)
COPD assessment test score	21 (5)
Total night sleeping time (min)	411 (72)
Wake time after sleep onset (min)	74 (36)
Sleep efficiency (%)	83 (8)

^a^COPD: chronic obstructive pulmonary disease.

### Continuous SpO _2_ Measurements, Preprocessing, and Data Quality

An overview of the amount of SpO_2_ measurements that were included for further analyses is provided in Table 2 and Multimedia Appendix 1. A total of 2 days and 2 nights of measurements from patient 4 were missing due to battery issues. A total of 3 days and 3 nights of measurements from patient 11 were excluded because correct time stamps were missing, due to the patient unintentionally resetting the time indication by taking out the batteries. The last day and night of patient 11’s measurements could not be analyzed because no clear distinction could be made between daytime and nocturnal data. Still, 3 days and 3 nights of patient 11’s measurements were used for further analyses.

**Figure 3:**
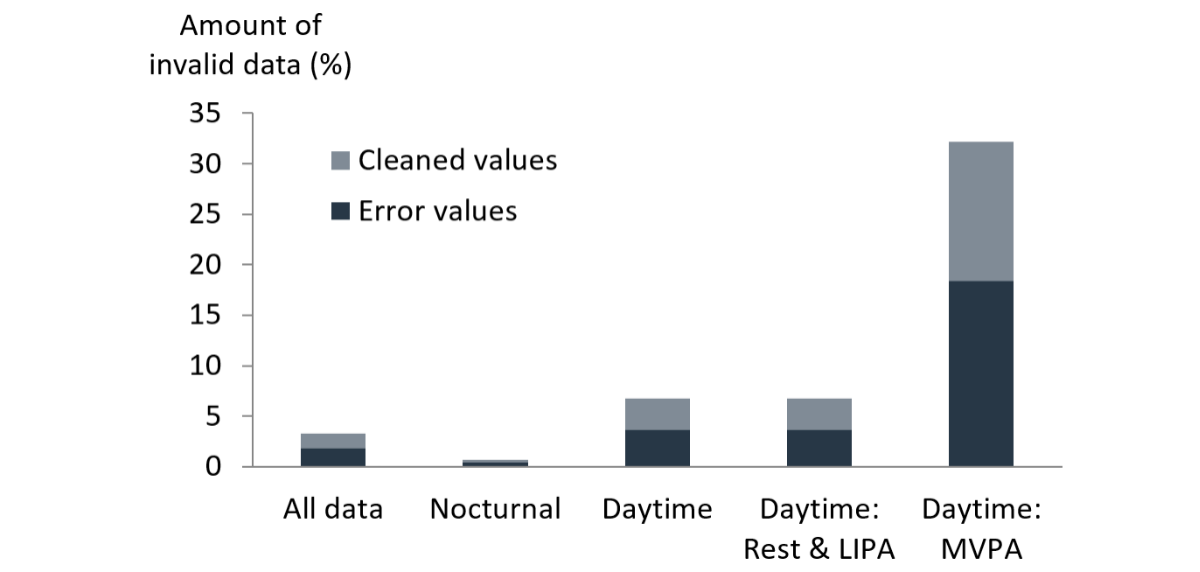
The amount of invalid data of continuous peripheral blood oxygen saturation (SpO_2_) measurements for different activities. LIPA: low-intensity physical activity; MVPA: moderate-to-vigorous physical activity.

The SpO_2_ dataset contained 1.83% error values and an additional 1.49% were excluded during the first two steps of the data-cleaning algorithm (see Figure 3), resulting in 96.68% of valid data. Nocturnal data had 99.31% of valid data (0.45% error data and 0.24% cleaned) compared to 93.27% of valid data during the day (3.62% error data and 3.11% cleaned). This was similar when only considering daytime data during rest and LIPA (EE≤3 MET; 93.90% of valid data: 3.25% error data and 2.85% cleaned). However, during MVPA (EE>3 MET), the amount of valid data decreased to 67.86% (18.34% error data and 13.80% cleaned).

**Table 2:** Summary of the amount of peripheral blood oxygen saturation (SpO_2_) measurements that were included for further analyses^a^.

Measurements included for further analyses		Mean (SD)
Days per patient		5.3 (1.8)
Nights per patient		5.9 (1.2)
**Hours per day**		
	Total	7.8 (3.9)
	During rest (EE^b^≤1.5 MET^c^)	6.1 (3.1)
	During LIPA^d^ (1.5 MET<EE≤3 MET)	1.5 (2.7)
	During MVPA^e^ (EE>3 MET)	0.2 (0.2)
Hours per night		8.0 (0.9)
**Data samples per patient**		
	Total	296,533 (95,580)
	During the day	149,665 (88,909)
	During the night	169,318 (35,488)

^a^All 20 patients performed nocturnal measurements and 17 patients performed daytime measurements. Therefore, averages were calculated with 17 patients for daytime measurements and 20 patients for nocturnal and total measurements.

^b^EE: energy expenditure.

^c^MET: metabolic equivalent of task.

^d^LIPA: low-intensity physical activity.

^e^MVPA: moderate-to-vigorous physical activity.

### SpO _2_ Features and Fluctuations

**Figure 4:**
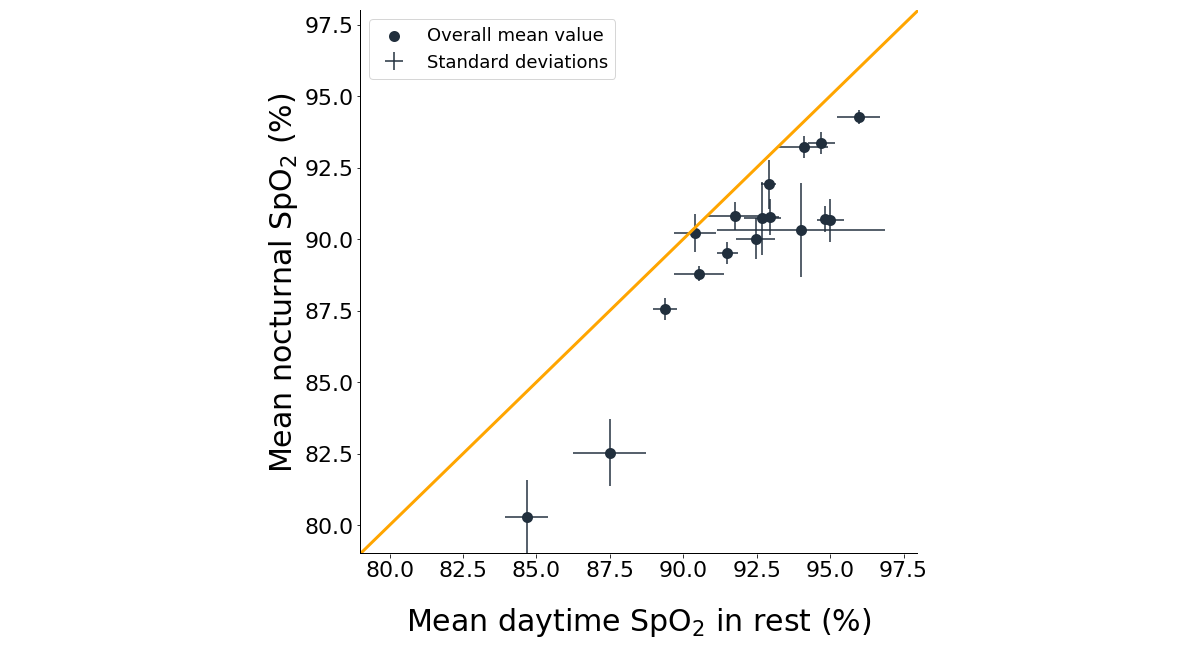
Mean nocturnal peripheral blood oxygen saturation (SpO_2_) compared to mean daytime SpO_2_ in rest. The dots indicate weekly averages of mean SpO_2_ for every patient, lines indicate the standard deviation of the mean SpO_2_ values over different days and nights, and the orange line is the line of equality.

The weekly averages of mean SpO_2_ were 89.9% (SD 3.4), 91.9% (SD 3.1), and 92.1% (SD 2.9) for nocturnal (n=20), daytime (n=17), and daytime-in-rest (n=17) measurements, respectively. Figure 4 shows that mean nocturnal SpO_2_ was lower than mean daytime SpO_2_ in rest (*P*<.001). Weekly averages of mean SpO_2_ were between 84.7% and 96.0% for daytime SpO_2_ in rest and between 80.3% and 94.3% for nocturnal SpO_2_ (see Figure 4). The weekly averages of CT90 were 35% (SD 34), 20% (SD 29), and 18% (SD 29) for nocturnal, daytime, and daytime-in-rest measurements, respectively. No significant correlations were found between patient characteristics and weekly averages of the SpO_2_ features.

Intraday and intranight SpO_2_ fluctuations were quantified as the weekly average of SpO_2_ SD for nocturnal (1.6%, SD 0.6), daytime (1.8%, SD 0.7), and daytime-in-rest (1.6%, SD 0.6) measurements. Nocturnal, daytime, and daytime-in-rest SpO_2_ SD values were inversely correlated with nocturnal, daytime, and daytime-in-rest mean SpO_2_ values (all: *P*<.001, R^2^>0.4), respectively. On average, SpO_2_ in rest values ranged over 10.8% (SD 4.4) within one day. There was a significant difference between mean SpO_2_ in the afternoon and in the evening (*P*<.001) (see Multimedia Appendix 3), with an average difference of 0.8% (SD 0.7) for those days where patients performed at least one hour of measurements during both the afternoon and evening. For this comparison, a total of 46 days from 12 different patients were used (median of 4 days per patient).

**Figure 5:**
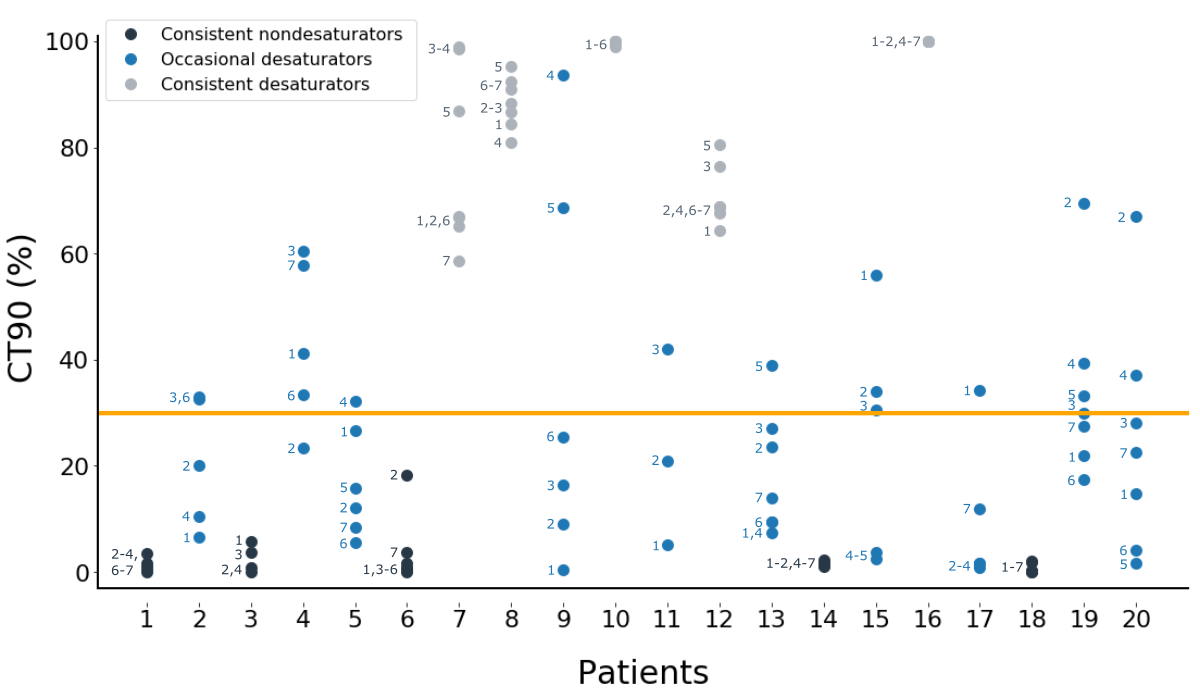
Cumulative time spent with peripheral blood oxygen saturation (SpO_2_) below 90% (CT90) values for every night of every patient showed that 50% (10/20) of the included patients changed category between desaturator and nondesaturator. The number next to each dot indicates the corresponding night of the measuring week and the orange line indicates the threshold of CT90 (30%) that divides nights with and without desaturation. A missing night number indicates that no data was available for that night.

Night-to-night and day-to-day SpO_2_ fluctuations were quantified as the weekly ranges of mean SpO_2_ (2.0%, SD 1.1; 1.9%, SD 1.5; 1.9%, SD 1.5) and CT90 (28%, SD 25; 19%, SD 20; 18%, SD 21) for nocturnal, daytime, and daytime-in-rest measurements, respectively. A more detailed analysis of the night-to-night changes in CT90 is shown in Figure 5. When considering the definition of a nocturnal desaturator, three types of patients can be distinguished: consistent desaturators (5/20, 25%), consistent nondesaturators (5/20, 25%), and occasional desaturators that changed category over the 7 nights (10/20, 50%). A total of 6 occasional desaturators (6/10, 60%) were desaturators or nondesaturators in the first two nights, but changed category when SpO_2_ measurements of another night were considered. The weekly average of mean SpO_2_ for occasional desaturators was between 89.5% and 91.9%, compared to 80.3%-88.8% and 91.8%-94.3% for consistent desaturators and consistent nondesaturators, respectively.

## Discussion

Continuous SpO_2_ measurements with wearable pulse oximeters identified significant SpO_2_ fluctuations between and within multiple days and nights of patients with COPD. COPD patients are known to be a heterogeneous group [32,33], which leads to large differences in mean SpO_2_ values between patients. Day-to-day SpO_2_ fluctuations have not yet received a lot of attention in scientific literature and night-to-night fluctuations have not yet been studied over more than two nights in patients with COPD. Some studies examined intraday fluctuations, however, these studies only examined short-term SpO_2_ fluctuations (ie, measurements of one hour or less) in healthy individuals [29] or infants [26-28]. Our results showed that significant SpO_2_ fluctuations occurred between and within multiple days and nights for the included patients. This is often not taken into account in telemonitoring applications [12]. When using the current definition of nocturnal desaturation, 50% (10/20) of the included patients changed category between desaturator and nondesaturator over the course of 1 week.

Continuous SpO_2_ measurements with high amounts of valid data could be obtained during a 7-day monitoring period, except during MVPA (EE>3 MET). Nocturnal measurements were well-perceived by the patients, leading to the high compliance. These measurements only had a small amount of data that was considered invalid (0.69%). This indicates that it is feasible to perform multi-night continuous SpO_2_ measurements. Most patients also performed daytime measurements during rest and LIPA (ie, 17 patients measured, on average, 7.6 hours per day). These had a limited amount of invalid data (6.10%). However, very few measurements were performed during MVPA (ie, 0.2 hours per day) and almost one-third of these measurements produced invalid data due to motion artifacts. Therefore, it might not be recommended to analyze continuous SpO_2_ measurements during periods of MVPA.

Two types of preprocessing steps for continuous SpO_2_ data have been reported: (1) removing deviating samples or (2) down-sampling the data. Deviating samples have been removed visually [29,34] by using an unspecified data-cleaning algorithm [35] or by removing samples with a sample-to-sample deviation of more than 4% [36] or 8% [37]. The latter method, however, does not remove artifacts that last over longer periods of time (eg, see Figure 2B: around 17:00-18:00). Therefore, artifacts in this study were removed by removing blocks of deviating values instead of separate samples. After sample removal, Morillo et al excluded remaining artifacts by down-sampling the data from 8 Hz to 1 Hz [37]. However, autocorrelation analyses in our study showed that a sampling time of 1 sample per second still led to oversampling of the data (see Multimedia Appendix 2). SpO_2_ could be down-sampled to 1 sample per 20 seconds for improved artifact removal, without losing information about the SpO_2_ dynamics.

Mean nocturnal SpO_2_ was lower than mean daytime SpO_2_ in rest. By taking into account the SpO_2_ fluctuations over different days and nights, the results of this study generalize the findings of Soguel Schenkel et al, who only performed SpO_2_ measurements during a single day and night that were up to five days apart [38]. Other studies used *awake* measurements of SpO_2_ to predict nocturnal mean SpO_2_ or CT90. They used the first 15-30 minutes of the nocturnal measurements to calculate the mean *awake* SpO_2_ values [15,39-41]. For these studies, it should be noted that slightly lower SpO_2_ values were observed in the evening compared to the afternoon, on average a 0.8% difference, and *awake* SpO_2_ values depend on the time of day when the measurements are performed. It is unlikely that this difference is solely due to the specified oximeter accuracy of ±2%, as no consistent bias has been reported for the WristOx_2_ when measuring over a longer time period.

The observed patient-to-patient, day-to-day, and intraday differences can have implications for telemonitoring applications that are based on daily SpO_2_ spot checks [12]. In several telemonitoring setups, alerts were raised when daily spot checks of SpO_2_ dropped below a generic threshold value (eg, 90% for all patients) [12]. However, the high patient-to-patient differences point out the shortcomings of these generic thresholds. Other telemonitoring setups used personalized thresholds, but these were still fixed on one specific threshold value for every patient [12]. For these setups, alerts can be merely a consequence of natural day-to-day and intraday fluctuations, instead of being triggered by the onset of an exacerbation. A recent paper better dealt with the day-to-day fluctuations by suggesting a day-to-day decrease of more than 4% to alert for exacerbations, however, a more thorough examination of this method is needed [42]. Intraday SpO_2_ values during rest ranged over 10.8% (SD 4.4) within one day. Moreover, statistical interpretation of weekly average of SpO_2_ SD (1.6%, SD 0.6), which is a measure of intraday fluctuations, indicates that during 5% of the day in rest, SpO_2_ fluctuates more than 3.2% (ie, 2 times SpO_2_ SD) beyond the mean daytime-in-rest value. In comparison, previous studies only reported a 1%-2% decrease in SpO_2_ spot checks around exacerbation onset compared to stable periods [43-45]. These natural intraday fluctuations can thus easily result in false alerts for exacerbations. Patients with lower SpO_2_ values might experience a higher number of false alerts as, similar to healthy individuals [29], intraday fluctuations (ie, SpO_2_ SD) increased with decreasing mean SpO_2_. Altogether, personalized alerts based on intelligent algorithms will be necessary to cope with all of these natural fluctuations in daytime SpO_2_ in rest. Preferably, these alerts should be based on continuous measurements over longer time periods, in contrast to the currently used daily spot checks, to account for the identified SpO_2_ fluctuations and to exclude the potential effect of the ±2% oximeter accuracy. In addition, the slightly lower SpO_2_ values in the evening compared to the afternoon show that the daily measurements should always be performed at the same moment of the day. A clearly defined measurement protocol, which is often not specified [12], can thus further attempt to limit the effect of natural SpO_2_ fluctuations in these telemonitoring applications.

The night-to-night differences of nocturnal mean SpO_2_ (ie, average weekly range of 2.0% over the different nights) led to highly varying CT90 values over the different nights of the measurement week (ie, average range of 28%). These high variations in CT90 resulted in 50% (10/20) of the included patients changing category between desaturator and nondesaturator, due to the fact that these occasional desaturators all had a mean SpO_2_ value around the threshold of 90%. A similar finding has been reported by Lewis et al, who concluded that 35% of the included patients changed category over two consecutive nights of measurements [20]. Later studies then tackled this problem by performing measurements over two nights, categorizing a patient as a desaturator if desaturation occurred in at least one of both nights [5,17-19]. However, the results of this study indicate that even two nights are insufficient to make a consistent separation between desaturators and nondesaturators, as 6 out of 10 occasional desaturators (60%) were desaturators or nondesaturators in the first two nights and only changed category afterward. Our results suggest that it might be impossible to consistently categorize COPD patients with a mean nocturnal SpO_2_ value around 90% as desaturator or nondesaturator. Based on measurements over multiple nights, these patients could thus be referred to as occasional desaturators. Further research is needed to assess the clinical relevance of identifying these three different nocturnal desaturation profiles.

Some limitations should be taken into consideration when interpreting the results of this study. The main limitation, as is often the case in similar studies, is the small sample size. Consequently, no comparison could be made between mild, moderate, and severe hypoxemic patients. Nevertheless, the increasing SpO_2_ SD with decreasing mean SpO_2_ suggests an increase in SpO_2_ fluctuations for more hypoxemic patients. No control group was included because this study aimed to perform continuous SpO_2_ measurements in COPD patients for identification of SpO_2_ fluctuations that could affect SpO_2_ applications, rather than comparing SpO_2_ between COPD patients and healthy controls. In addition, daily home routines can greatly differ between patients and healthy controls [46], impeding a proper comparison. As shown in Multimedia Appendix 1, daytime measurements were only seldom performed during full days due to the impracticalities of wearing the finger clip during activities that require manual actions (eg, during morning routines). This, in combination with the high amount of invalid data during MVPA, suggests that continuous SpO_2_ measurements with a finger clip will have more profound limitations in a population that is more physically active than the target population of this study. Reliable wearable oximeters that do not require a finger clip could thus increase compliance. Moreover, the limited battery life and the inability of real-time data transmission of the used oximeter can further complicate the integration of continuous SpO_2_ measurements into practice. Technological advances are thus needed to allow for long-term, continuous monitoring of SpO_2_. This study, however, mainly intended to show the potential of prolonged continuous measurements to identify SpO_2_ fluctuations. Therefore, a certified wearable pulse oximeter with finger clip was preferred over more user-friendly, watch-type oximeters that have not yet been proven to be accurate. The resulting, more fragmented, daytime measurements were sufficient to identify large natural fluctuations occurring within one day (ie, SpO_2_ in rest ranged over 10.8% [SD 4.4] within one day), confirming the a priori posed hypothesis. The limited battery life was addressed by providing the patients with multiple sensors to cover the 7-day monitoring period.

This study was the first to use wearable finger pulse oximeters for prolonged continuous SpO_2_ measurements in COPD patients, as opposed to only performing spot checks or continuous measurements during one or two nights. These measurements showed that spot checks or one- or two-night measurements should be interpreted with caution, as the conclusions based on these measurements might change depending on the moment of the measurement. Measurements were performed at home during daily life routines of COPD patients, which provides a more natural SpO_2_ profile compared to supervised measurements. By adding actigraphy measurements, the necessary contextual information could be gathered for more accurate analyses of the continuous SpO_2_ measurements.

In conclusion, continuous SpO_2_ measurements with wearable pulse oximeters identified significant SpO_2_ fluctuations between and within multiple days and nights of patients with COPD. Continuous SpO_2_ measurements during the daily home routine of patients with COPD generally had high amounts of valid data, except for motion artifacts during MVPA. The continuous measurements showed that mean nocturnal SpO_2_ was lower than mean daytime SpO_2_ in rest, and significant SpO_2_ fluctuations occurred between and within multiple days and nights. The large fluctuations of daytime SpO_2_ in rest indicate that clear measurement protocols and personalized alerts, based on intelligent algorithms, will be needed to increase the performance of telemonitoring applications that make use of daily SpO_2_ spot checks. Lastly, it was shown that CT90 values can vary greatly from night to night in patients with a nocturnal mean SpO_2_ around 90%, indicating that these patients cannot be consistently categorized as desaturators or nondesaturators. We recommend using wearable sensors for performing continuous SpO_2_ measurements over longer time periods to determine the clinical relevance of the identified SpO_2_ fluctuations.

## Appendix

Multimedia Appendix 1 Visual overview of the continuous peripheral blood oxygen saturation (SpO2) measurements that were included for analyses.

Multimedia Appendix 2 Autocorrelation analyses.

Multimedia Appendix 3 Differences between mean peripheral blood oxygen saturation (SpO2) in the afternoon and in the evening. Grey dots indicate the differences within the same day for the same patient and orange dots indicate the average of these differences for every patient. The number of grey dots per patient indicate the amount of days with measurements in both the afternoon and evening.

## Abbreviations

6MWD: 6-minute walking distance
CAT: chronic obstructive pulmonary disease assessment test
CIRO: Centre of Expertise for Chronic Organ Failure
COPD: chronic obstructive pulmonary disease
CT90: cumulative time spent with SpO_2_ below 90%
EE: energy expenditure
FEV1: forced expiratory volume in 1 second
FVC: forced vital capacity
GOLD II-IV: Global Initiative for Chronic Obstructive Lung Disease stages II-IV
ISO: International Organization for Standardization
LIPA: low-intensity physical activities
MEC-U: Medical Research Ethics Committees United
MET: metabolic equivalent of task
MVPA: moderate-to-vigorous physical activities
PaO _2_: oxygen partial pressure in arterial blood
Seff: sleep efficiency
SpO _2_: peripheral blood oxygen saturation
TLCO: transfer factor for carbon monoxide
TNST: total night sleeping time
VITO: Flemish Institute for Technological Research
WASO: wake time after sleep onset
