# Supplementary figures and images for: Wearable Finger Pulse Oximetry for Continuous Oxygen Saturation Measurements During Daily Home Routines of Patients With Chronic Obstructive Pulmonary Disease (COPD) Over One Week: Observational Study
Source: JMIR Mhealth Uhealth. 2019 Jun 6;7(6):e12866. doi: 10.2196/12866 (PMC6594211; doi:10.2196/12866)

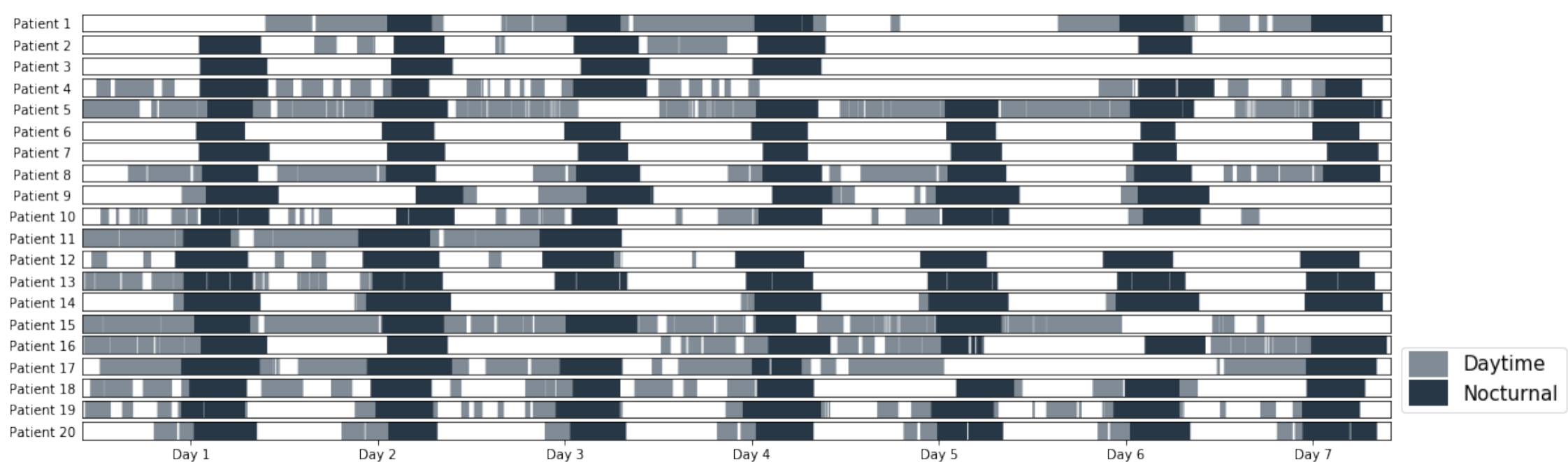

Supplement: Multimedia Appendix 1 [file mhealth_v7i5e12866_app1.pdf]

Patient

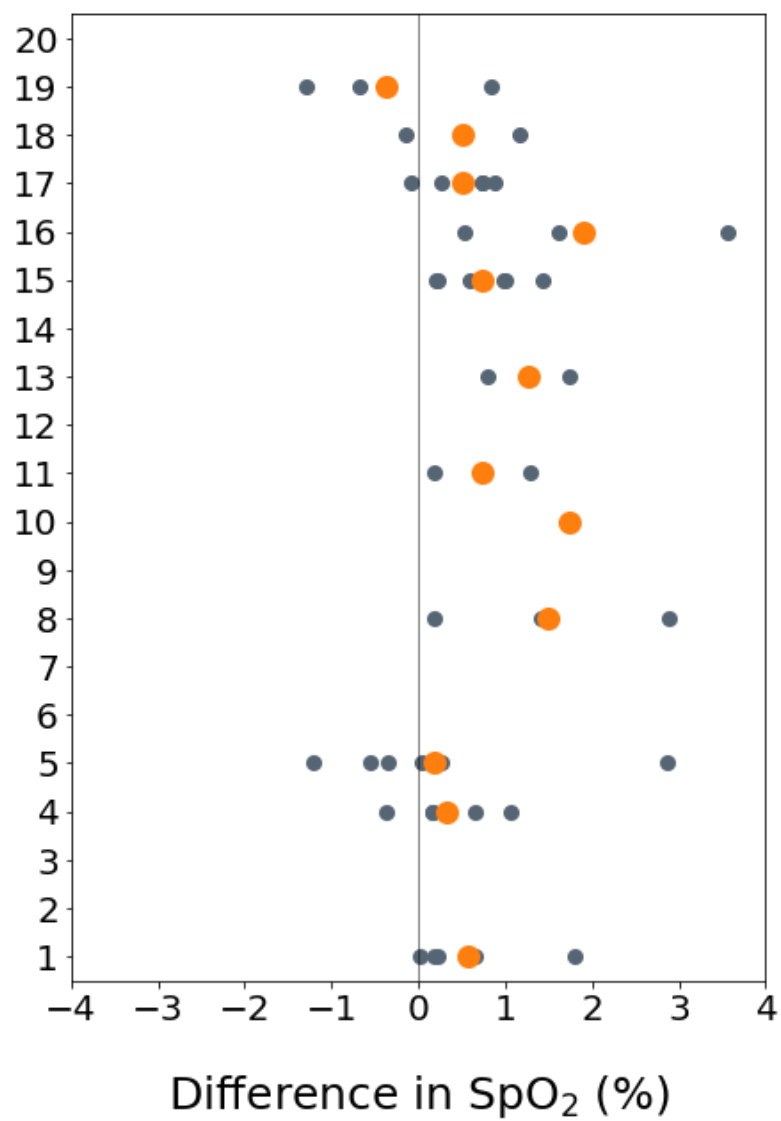

Supplement: Multimedia Appendix 3 [file mhealth_v7i6e12866_app3.pdf]
