# Supplementary material for: Wearable Finger Pulse Oximetry for Continuous Oxygen Saturation Measurements During Daily Home Routines of Patients With Chronic Obstructive Pulmonary Disease (COPD) Over One Week: Observational Study
Source: JMIR Mhealth Uhealth. 2019 Jun 6;7(6):e12866. doi: 10.2196/12866 (PMC6594211; doi:10.2196/12866)
Supplement: Multimedia Appendix 2 [file mhealth_v7i6e12866_app2.pdf]

Multimedia Appendix 2

Autocorrelation analyses were performed to test whether SpO_2 data could be down-sampled without losing information about the dynamics of the SpO_2 signal. For every patient, an autocorrelation value with lag 1 (AR(1); i.e. the correlation between two consecutive values) was calculated for different sampling times ranging between 1 and 100 seconds, based on all their available SpO_2 data. Hereafter, the average AR(1) value of all patients was calculated for every sampling time (see figure). AR(1) of the data at the original sampling time of 1 second was 0.99 (± 0.01), indicating consecutive values are highly correlated and the time series could be down-sampled without losing information about the dynamics of the data. Resampling the data with increasing sampling times led to a quick drop in AR(1) to 0.81 (± 0.09) at a sampling time of 20 seconds, after which AR(1) decreased more steadily to 0.73 (± 0.10) at a sampling time of 100 seconds (see figure). This initial drop in AR(1) was due to the diminished effect of oversampling. Therefore, the turning point at 20 seconds was chosen as the best suiting sampling time for improved artefact removal without losing information about the SpO_2 dynamics.

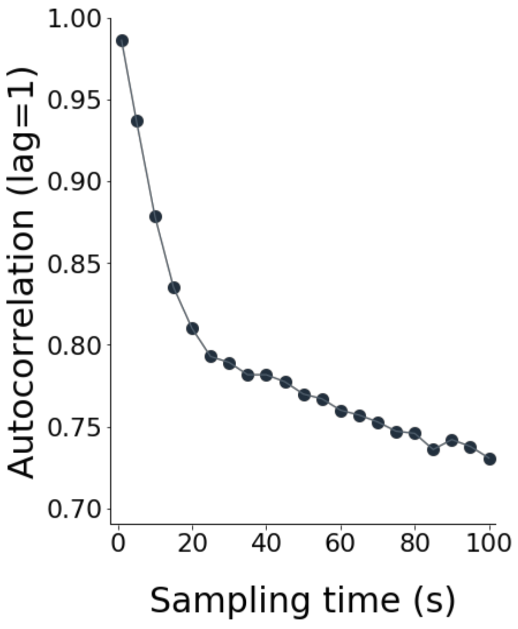

Autocorrelation with lag 1 for sampling times ranging between 1 and 100 seconds.
